# The Physiology and Cure of Stuttering

**Published:** 1895-06-22

**Authors:** 


### The Hospital

An Institutional Journal of

The Medical Sciences and Hospital Administration, with

AN EXTRA NURSING SUPPLEMENT.

Vol. XVIII.—No. 456.] [June 22, 1895.

### The Physiology and Cure of Stuttering.

Stuttering, which is often so embarrassing to the victim of it, is a very simple business when physiologically analysed. Dr. W. S. Colman has made a praiseworthy attempt to bring the abnormality in from the outer darkness of unauthorised specialism. There would appear, he says, to be no more reason why stuttering should be left to the tender mercies of the non-medical pretender than why any ordinary "surgical" disease should be treated by the instrument maker. The only reason, we would suppose, why stammering and stuttering have been so long handed over to the quack is that the orthodox medical man has considered them such insignificant and yet such intractable abnormalities that he has not had sufficient patience to deal practically with them.

What is the mechanism of stammering and stuttering, or rather of "stuttering," as Dr. Colman prefers that we should alone call the process? The stutterer may be called one of Nature's failures, for Nature is by no means always perfect in the results of her working: very far from it. Into normal voice and articulation three main factors enter, and it is essential for the production of perfect speech that these three factors be put in operation simultaneously. The want of complete power to put all three in action simultaneously is the cause of stuttering. The three factors are: the respiratory apparatus, which must supply the blast of wind needed for the production of sound; the larynx, which converts the blast into definite sound; and the musculature of the tongue, lips, and palate, which makes out of mere sound articulate words. All these three factors are put in operation, each by its own set of muscles, and the muscles in their turn are operated by discharges of nerve energy. If the discharges of nerve energy act simultaneously upon all three sets of muscles, and if they are of adequate force to bring all three into due operation at one and the same time, normal voice and speech are the unvarying result. It is conceivable that in some rare instances that the muscular apparatus of any one of the factors may itself be the faulty part. But in the vast majority of cases of stuttering it is the nervous energy which is irregularly supplied, and stuttering, in ultimate analysis, is a nervous, or a nervo-muscular abnormality.

Whilst the physiology of the process as well as the cure of the abnormality is interesting to medical men, the laity will not unnaturally be much more interested in the cure. Is it really possible to cure stuttering? Undoubtedly it is in many cases, and in most a considerable amount of improvement may be effected. But the cure of stuttering is like "learning," there is no "royal road" to it. Time, patience, intelligence; intelligence, patience, time; these and these alone are the indispensable conditions of cure. It is to be remembered above all things that whilst the doctor can guide and show the way, it is the patient himself who must effect the cure. Given patients of average intelligence and firm will, there are very few cases which cannot be cured or greatly improved. The first requisite is a determination to follow instructions in every detail. As to the many details, Dr. Colman tells us that the chest should be well filled with air before any attempt is made to speak; and it should be deliberately refilled and kept full as long as speech is continued. The blast of air, which is the first of the three conditions of perfect speech, is thus kept always strong and always in motion. If a patient has a poor chest capacity he should make every effort to increase it by dumb-bell and other chest-expanding exercises. Another essential condition is to habitually speak in a clear, resonant voice. Mumbling, whispering, and low speech are great incentives to stuttering. When these and other details have been learnt by heart, and have been repeatedly put in practice under the trained supervision of the physician; the rest is usually comparatively easy. The one word "practice" sums up the final condition of complete success.

The study of the subject of stuttering is by no means so limited or uninteresting as might be supposed. In the very brief space at our disposal we are under the necessity of confining ourselves almost entirely to leading principles, and to those in their most meagre and skeletal forms. Dr. Colman has gone much deeper. He tells us, for example, that stuttering is twice as common among males as among females. Yet it is a well-known fact that the nervous apparatus of the woman is much more sensitive than that of the man. The abnormality is, moreover, world-wide; the negro races of Central

Africa and the Mongolians of China suffering from it as widely as the English, or the still more nervous French. Stuttering, moreover, whilst not itself necessarily a sign of defective mental capacity, is nevertheless a frequent associate of limited mental endowment, and is especially common among epileptics. These and other like facts render it imperative that stutterers be dealt with, not by persons of no scientific competency, who can only apply, without intelligence, mere crude "rules of thumb," but by educated physicians, who know exactly the seat and intimate nature of the disorder, and who can apply scientific remedies to ills scientifically investigated.

